# Beatrice Hill Virus Represents a Novel Species in the Genus *Tibrovirus* (*Mononegavirales*: *Rhabdoviridae*)

**DOI:** 10.1128/genomeA.01485-16

**Published:** 2017-01-26

**Authors:** Michael R. Wiley, Karla Prieto, Kim R. Blasdell, Yíngyún Caì, Cristine Campos Lawson, Peter J. Walker, Charles Y. Chiu, Gustavo Palacios, Jens H. Kuhn

**Affiliations:** a U.S. Army Medical Research Institute of Infectious Diseases, Fort Detrick, Frederick, Maryland, USA; b Commonwealth Scientific and Industrial Research Organization (CSIRO), Health and Biosecurity, Australian Animal Health Laboratory, Geelong, Victoria, Australia; c Integrated Research Facility at Fort Detrick, National Institute of Allergy and Infectious Diseases, National Institutes of Health, Fort Detrick, Frederick, Maryland, USA; d School of Biological Sciences, University of Queensland, St. Lucia, Queensland, Australia; e University of California, San Francisco, California, USA

## Abstract

The rhabdoviral genus *Tibrovirus* currently has three official members assigned to two species: Bivens Arm virus and Tibrogargan virus (species *Tibrogargan tibrovirus*) and Coastal Plains virus (species *Coastal Plains tibrovirus*). Here, we report the complete genome sequence of a new putative member of this genus, Beatrice Hill virus. Although relatively closely related to the three classified viruses, Beatrice Hill virus represents a novel *Tibrovirus* species.

## GENOME ANNOUNCEMENT

The mononegaviral family *Rhabdoviridae* currently includes 13 genera and four unassigned species (1). One of these genera, *Tibrovirus*, includes two species: Bivens Arm virus (BAV) and Tibrogargan virus (TIBV) have been assigned to the species *Tibrogargan tibrovirus*, and Coastal Plains virus (CPV) has been assigned to the species *Coastal Plains tibrovirus* (1–3). TIBV and BAV were originally isolated from biting midges (*Culicoides brevitarsis* and *C. insignis*, respectively), and CPV from an apparently healthy steer (4–6). Recently, several novel viruses have been identified as putative tibroviruses. These include (a) Bas-Congo virus (BASV) in the serum of a human with viral hemorrhagic fever (7), (b) Ekpoma virus 1 (EKV-1) and Ekpoma virus 2 (EKV-2) in sera of apparently healthy humans (8), and (c) Sweetwater Branch virus (SWBV) in biting midges (*Culicoides insignis*) (3, 6). These viruses have recently been accepted by the International Committee on Taxonomy of Viruses (ICTV) to represent the novel species *Bas Congo tibrovirus*, *Ekpoma 1 tibrovirus*, *Ekpoma 2 tibrovirus*, and *Sweetwater Branch tibrovirus*, respectively (9). All tibroviruses have the specific genomic structure 3′-N-P-M-U1-U2-G-U3-L-5′, with N-P-M-G-L being canonical rhabdoviral genes encoding structural proteins and U1, U2, and U3 being tibrovirus-unique genes. U1 and U2 encode proteins of unknown function, and U3 encodes a protein with the structural characteristics of a viroporin (3, 9, 10).

Beatrice Hill virus (BHV) was first reported in 1984 as a novel virus of biting midges (*Culicoides peregrinus*) that had been collected at Beatrice Hill, Northern Territory, Australia (11). In 2016, Huang et al. reported a 5,734 nt-long contig of the BHV genome, which indicated that this virus most likely falls into the *Tibrovirus* clade (12). To determine the taxonomic position of BHV, we obtained a historical sample of brain tissue from a laboratory mouse that had been infected intracranially with BHV-containing material. Viral RNA from this sample was processed and sequenced to obtain the complete genome using a sequence-independent single-primer amplification (SISPA) protocol that included rapid amplification of cDNA ends (RACE) (13). Resulting libraries were sequenced on an Illumina MiSeq desktop sequencer. Illumina and SISPA-RACE adapter sequences were trimmed from the sequencing reads using Cutadapt version 1.2.1 (14), quality filtering was conducted with Prinseq-lite (15), and reads were assembled into contigs using Ray Meta with a *k*-mer length of 25 (16). Resultant contigs were aligned to the National Center for Biotechnology Information (NCBI) sequence database using BLAST. Reads were aligned back to the assembled BHV sequence using Bowtie2 (17), and custom scripts were used to generate a final consensus sequence.

Using PASC (18), the determined complete BHV genome sequence was found to be 72.79% identical to that of TIBV (GenBank accession no. GQ294472.1) and 72.48% identical to that of BAV (KP688373.1). The genome organization matches that of all tibroviruses, therefore confirming Huang et al.’s hypothesis that BHV is a tibrovirus. Our data furthermore suggest that this virus should be assigned to a novel species (“*Beatrice Hill tibrovirus*”) based on its divergence from TIBV and BAV.

### Accession number(s).

The GenBank accession number of Beatrice Hill virus isolate CSIRO 25 is KY073493.
